# Methodological Approaches to Dengue Virus Detection in Wastewater: A Systematic Review and Meta-Analysis of Positivity Rate

**DOI:** 10.3390/v18050531

**Published:** 2026-04-30

**Authors:** Siti Aishah Rashid, Sakshaleni Rajendiran, Nurul Farehah Shahrir, Nurul Athirah Naserrudin, Terence Tan Yew Chin, Janice Chan Sue Wen, Imanul Hassan Abdul Shukor, Nurul Amalina Khairul Hasni

**Affiliations:** 1 Environmental Health Research Centre, Institute for Medical Research, National Institutes of Health, Ministry of Health, Shah Alam 40170, Malaysia; sakshaleni@moh.gov.my (S.R.); farehah.shahrir@moh.gov.my (N.F.S.); drn.amalina@moh.gov.my (N.A.K.H.); 2 Institute for Health Systems Research, National Institutes of Health, Ministry of Health, Shah Alam 40170, Malaysia; nurulathirah.n@moh.gov.my; 3 Integrative Health Information Unit, Herbal Medicine Research Centre, Institute for Medical Research, National Institutes of Health, Ministry of Health, Shah Alam 40170, Malaysia; terencetyc@moh.gov.my (T.T.Y.C.); janice.cs@moh.gov.my (J.C.S.W.); 4 Institute for Health Management, National Institutes of Health, Ministry of Health, Shah Alam 40170, Malaysia; imanul@moh.gov.my

**Keywords:** dengue virus, wastewater-based epidemiology, wastewater, community surveillance, spiked-in study

## Abstract

Dengue fever, with a high proportion of asymptomatic infections, poses a major global public health challenge that traditional surveillance systems frequently underestimate. Wastewater-based epidemiology (WBE) has emerged as a promising approach to monitoring infectious diseases beyond enteric viruses. Dengue virus is shed in urine, feces, and saliva, providing a biological basis for wastewater detection alongside clinical surveillance. This systematic review and meta-analysis synthesize current evidence on dengue virus (DENV) detection in wastewater and evaluate methodological factors influencing detection success in WBE. A systematic literature search using selected databases and predetermined keywords, followed by eligibility screening, resulted in ten studies being included, covering community surveillance and experimental trials. DENV ribonucleic acids (RNA) were most consistently detected and enriched in wastewater solids, indicating this matrix as the most reliable for surveillance. Among concentration methods, ultrafiltration achieved the highest viral recovery efficiency, while reverse transcription digital polymerase chain reaction (RT-dPCR) demonstrated superior sensitivity and precision compared to those of reverse transcription quantitative polymerase chain reaction (RT-qPCR), particularly at low viral concentrations. Storage at −80 °C was critical for preserving RNA integrity. The meta-analysis yielded a pooled DENV positivity rate of 24% (95% CI: 20–28%) after exclusion of outliers. Overall, solid-phase analysis combined with RT-dPCR represents the most sensitive methodological approach across the included studies. Harmonized protocols are needed to support future translation of dengue WBE into community surveillance as current evidence mainly demonstrates methodological feasibility and provides a technical foundation for future public health integration. Therefore, further longitudinal and multi-site validation is required to establish its broader applicability for dengue surveillance.

## 1. Introduction

Dengue virus (DENV) represents an escalating global public health threat due to its expanding geographic range and rising incidence. The virus is now endemic in over 100 countries and is estimated to cause 390 million infections annually, the majority of which are asymptomatic [1,2]. Epidemiological data estimates indicate that 50–80% of dengue infections are asymptomatic or subclinical, presenting with absent or mild, non-specific symptoms that often evade clinical detection due to missed diagnosis or low healthcare-seeking behavior [3,4]. Both symptomatic and asymptomatic individuals contribute to DENV transmission; however, asymptomatic infections complicate surveillance due to limited detection through routine clinical reporting systems [5,6]. Given that dengue can spread silently through asymptomatic individuals, and traditional surveillance relies mainly on clinically reported cases, the true prevalence of DENV infections is often underestimated, making early detection of outbreaks more challenging.

To address these limitations, WBE has emerged as a potential and non-invasive complementary tool for population-level disease monitoring [7,8]. WBE has proven effective in tracking enteric and respiratory pathogens, including poliovirus and severe acute respiratory syndrome coronavirus 2 (SARS-CoV-2). This approach relies on detecting ribonucleic acid (RNA) shed into wastewater by both symptomatic and asymptomatic carriers, contributing data that complements clinical case reporting [6,9,10]. DENV can be detected in bodily fluids such as urine and feces from both symptomatic and asymptomatic individuals, as demonstrated in previous studies, supporting its feasibility for wastewater surveillance [11,12]. Correlation between wastewater signals and clinical case trends for other arboviruses like Zika virus (ZIKV) and Japanese encephalitis virus (JEV) further highlights its potential as a complementary surveillance approach [13,14].

Several reviews on arboviruses WBE contribute valuable insights, yet systematic synthesis of applications for dengue WBE remains limited. One study assessed key factors affecting feasibility including viral shedding, persistence, and recovery efficiency [15], while another study highlighted methodological challenges and advances in detecting yellow fever virus and related arboviruses [13]. In contrast, a recent article cautions that applying WBE to vector-borne diseases like dengue and malaria requires careful consideration of pathogen biology, geographic setting, and sewage infrastructure [16]. Despite its potential, the relative significance of dengue WBE remains insufficiently studied. The major bottleneck is the substantial dilution of DENV in wastewater, which requires a robust and intelligent concentration methods to obtain reliable signals. Variability in detection rates, coupled with the lack of standardized and optimized protocols, further complicates interpretation. Consequently, there is no consensus on the most effective methodological approach at present.

Currently, no systematic review has compiled methodological factors of DENV detection in wastewater or quantified pooled detection success across diverse settings. Therefore, we aim to examine methodologies and key findings related to dengue WBE while highlighting critical knowledge gaps. It focuses on describing variability in current methodologies, measuring DENV positivity rate in wastewater to assess methodological feasibility, and outlining the implications for future public health applications.

## 2. Materials and Methods

This review was conducted and reported following the Preferred Reporting Items for Systematic Reviews and Meta-Analyses (PRISMA 2020) Statement [17]. The PRISMA checklist is included in Supplementary Table S1. This review was registered in the International Prospective Register of Systematic Reviews (PROSPERO) with the ID: CRD 42024574930 and the protocol can be accessed at https://www.crd.york.ac.uk/PROSPERO/view/CRD42024574930, accessed on 20 April 2026.

### 2.1. Identifying the Research Questions

The research questions guiding this review include:

1. What methodologies and key findings have been reported in studies on dengue WBE?
2. What is the reported prevalence or concentration of DENV detected in wastewater according to existing studies?
3. What critical knowledge gaps exist in current research on dengue WBE?
4. How can dengue WBE be applied to support or guide public health actions?

### 2.2. Identifying Relevant Studies

A systematic search of PubMed, Scopus, Web of Science (WOS), and Embase was carried out by two independent reviewers following the predefined eligibility criteria. The search strategy included a combination of keywords comprising Medical Subject Heading (MeSH) terms and free-text terms related to “dengue” and “wastewater-based epidemiology”. Boolean operators such as OR and AND were used to combine these keywords and refine the search findings in the titles and abstracts of the databases. Additionally, a hand search with manual screening of references from articles was performed. The time frame for the inclusion of studies was from inception until October 2025. All search results were screened and reported in accordance with the PRISMA (Preferred Reporting Items for Systematic Reviews and Meta-Analyses) guidelines. Full details of the search strategy are available in Supplementary Table S2.

### 2.3. Study Selection and Eligibility Criteria

This review included peer-reviewed studies with descriptive, experimental, longitudinal, time-series, or cross-sectional designs that investigated the presence of DENV ribonucleic acid (RNA) in wastewater. Only articles published in English and available in full text were considered. Eligibility criteria were defined using the PICO framework.

#### 2.3.1. Inclusion Criteria

- Population: Wastewater or sewage samples collected from waste-water treatment plants (WWTPs) serving community catchments, including resident area and institutional settings within sewered regions.
- Intervention: WBE, including variations in sampling approaches (e.g., sample type, collection technique, frequency, and location), detection methods, and serotype analysis.
- Comparator: Variations in methodological approaches; studies were included regardless of whether a comparator group was reported.
- Outcome: Viral load measurements (e.g., gene copies and cycle threshold values (Ct-Value) assessed across different contexts, including sampling methodologies, detection methods (PCR-based, ELISA), and geographical regions. In addition, studies reporting serotype analysis, positivity rates (%), epidemiological trends (e.g., above or below thresholds, stagnation, increase, or decrease in case numbers), and molecular characterization of DENV were also included.

#### 2.3.2. Exclusion Criteria

Excluded documents included:

- Reviews, newspaper articles, commentaries, editorials, pre-prints, conference abstracts, case reports, technical reports, books or book chapters
- Non-English publications
- Studies involving in vivo or in vitro experiments on animals, cells, or tissues were also excluded.

#### 2.3.3. Screening

**Figure 1:**
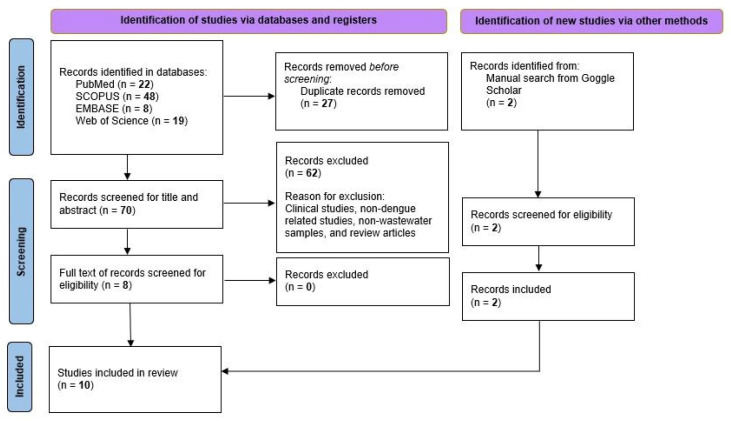
Flowchart of search strategy based on the PRISMA (Preferred Reporting Items for Systematic Reviews and Meta-Analyses) protocols.

Preliminary title and abstract screening of the remaining articles was conducted independently by two reviewers based on the predetermined PICO criteria. Subsequently, full-text articles were independently assessed for eligibility using predefined inclusion and exclusion criteria. Inter-reviewer agreement was assessed using percent agreement, which demonstrated a high level of concordance at both the title/abstract screening stage and the full-text screening stage. Reasons for exclusion at the full-text stage were discussed and recorded. The study selection process was documented using a PRISMA flow diagram, as shown in Figure 1. Articles retrieved from electronic searches were imported into Zotero software (version 8.0.3) for deduplication and subsequently exported to Microsoft Excel for screening and data management.

### 2.4. Data Extraction

An extensive data extraction was applied using a standardized form to systematically gather information from each study. This included:

1. Study characteristics (author details, publication year, country, study design);
2. Sampling methodology (sample type, sampling techniques, frequency, location);
3. Method of detection;
4. Serotype analysis;
5. Viral load concentrations and positivity rate.
6. Limits of detection/quantification (LOD/LOQ)

Positivity definition is standardized according to the total number of samples collected in each study. Where reported, the limits of detection (LOD) and limits of quantification (LOQ) were also extracted. To ensure the reliability of the data, two independent reviewers performed the extraction, with discrepancies resolved through discussion or consultation with a third reviewer. This allowed for a comprehensive evaluation of the evidence, highlighted significant findings, pinpointed research gaps, and offered a foundation for future studies in the field. The standardized data extraction form template can be found in Supplementary Table S3.

### 2.5. Risk of Bias Assessment (RoB)

The Risk of Bias in Non-randomized Studies of Interventions (ROBINS-I) tool, which evaluates the risk of bias and the effect of an intervention on an outcome from individual non-randomized study, was used to assess the quality of the included studies [18]. This tool evaluated the risk of bias across seven domains: (i) bias due to confounding, (ii) bias in the classification of interventions, (iii) bias in the selection of participants into the study (or into the analysis), (iv) bias due to deviations from intended interventions, (v) bias due to missing data, (vi) bias arising from measurement of the outcome, and (vii) bias in the selection of the reported result. Each domain was rated as having low, moderate, serious, or critical risk of bias. Two independent reviewers evaluated all domains, and any inconsistencies were resolved through consultation by a third reviewer. The overall risk of bias for each study was determined using the “worst-score rule,” whereby the final judgement reflected the highest level of bias identified in any single domain. For the RoB assessment, the results for each study were tabulated using Microsoft Excel to allow clear comparison across domains.

### 2.6. Statistical Analysis

Statistical analysis was conducted using STATA V18.0. A meta-analysis was performed to determine the overall positivity rate, generating pooled proportions with corresponding 95% confidence intervals (CIs). Heterogeneity was evaluated using the I^2^ statistic. If I^2^ exceeded 80%, a random-effects model was applied; otherwise, a fixed-effects model was used [15]. Subgroup analyses were performed to explore potential sources of heterogeneity. Sensitivity analysis was undertaken using the leave-one-out approach, in which individual studies were sequentially excluded to evaluate their influence on the pooled estimate and heterogeneity. Statistical significance was defined as *p* < 0.05. For the meta-analysis, pooled estimates and individual study results were visually displayed using forest plots generated in STATA.

## 3. Results

### 3.1. Summary of Characteristics of Included Studies

A comprehensive search across four databases identified 97 articles relevant to the objective of this systematic review. Upon screening of title and abstract followed by full-text screening based on eligibility assessment, ten studies were selected for inclusion. Figure 1 details the screening process based on PRISMA protocols.

Despite the broad timeline considered, the number of eligible studies remained low, with the majority being published after the year 2020, corresponding to the onset of the Coronavirus disease (COVID-19) pandemic. Table 1 summarizes the studies included in this review. The geographical distribution of the included studies was observed across eight countries; the USA (n = 2), Singapore (n = 2), Taiwan (n = 1), Nepal (n = 1), Brazil (n = 1), Portugal (n = 1), China (n = 1), and Italy (n = 1). This review classifies the included studies into two main groups: population-based surveillance studies and experimental spiking studies. Sampling sites ranged from WWTPs to manholes, covering populations from 100 to over 1,000,000 people. Both grab and composite sampling were employed, with collection volumes ranging from 30 to 1000 mL. Samples were commonly transported at 4 °C, while long-term storage of viral RNA and other materials was carried out at −25 °C or −80 °C to preserve sample integrity for downstream analyses.

### 3.2. Overview of Population and Experimental-Based Approaches in Dengue WBE

Six of the studies included directly investigated population-based wastewater surveillance aiming to detect DENV RNA and monitor dengue outbreaks [16,17,18,19,20,21] and to evaluate the potential use of WBE as a complementary tool to strengthen clinical surveillance [22,24]. Two studies [21,24] specifically focused on the detection of DENV RNA in wastewater solids, compared to other studies using raw wastewater samples. Only one study included dengue among other focal viruses to demonstrate the application of genomic surveillance for population-level monitoring [19]. The remaining four studies [22,23,24,25] investigated virally spiked wastewater to characterize viral persistence and viral decay while also optimizing techniques for clarification, concentration method and extraction methods [25,27,28]. This gives insights on the stability of the virus at various environmental conditions. A study directly examined the decay kinetics of DENV, ZIKV, and yellow fever viruses in untreated wastewater [26]. Recent works compared different viral recovery methods to conclude about the superior techniques for optimal workflow [25,27]. Another study explored the partitioning behaviors of several arboviruses, including DENV, and emphasized the advantages of using solids as the matrix in a low-prevalence setting [28].

### 3.3. Detection Rate Outcomes and Methodological Evaluation in Population-Based Dengue WBE Studies

Detection of DENV RNA in wastewater has been reported in multiple surveillance studies, with positivity rates varying by region, outbreak intensity, and methodological factors. The highest detection rate 33.3% was reported in Central Italy [21] during the decline of a local outbreak, followed by 30.8% in hospital sewage and 20% in municipal wastewater samples in Brazil between February and March 2023, coinciding with an increase in reported dengue cases from 776 to 2874 [19]. In Portugal, DENV RNA was detected in 25% of samples collected from 11 treatment plants between May 2022 and April 2023, with two seasonal peaks observed in September 2022 and February–April 2023 [22]. A 21% positivity rate was observed in Florida between June and September 2023, aligning with clinical findings that indicate the predominance of a single circulating serotype (DENV-3) [24]. Conversely, targeted population-level monitoring in Guangzhou, China revealed a low detection rate of 2.3% [20], while Nepal reported the absence of a measurable signal despite widespread wastewater testing [23].

Detection success in WBE was influenced by the sampling approach, sample type, concentration method, and molecular detection technique. Studies consistently showed that wastewater solids provided higher viral enrichment compared to that in liquid fractions, highlighting the importance of sample type selection. Various concentration methods were employed, including ultrafiltration, magnetic beads, hollow-fiber devices, and electronegative membrane filtration, with recovery efficiency varying across studies. For instance, in Central Italy (Marche Region, 2024) Mancini et al. reported that four concentration methods were evaluated; however, DENV RNA was detected exclusively in the solid fraction, with RT-dPCR showing higher sensitivity detecting 9 of 27 samples (33.3%) than real-time RT-PCR identifying 5 of 27 samples (18.5%) [21]. In Brazil, electronegative membrane filtration combined with RT-qPCR and hybrid capture sequencing enabled whole-genome DENV-1 identification [19].

In Portugal, Monteiro et al. utilized hollow-fiber ultrafiltration with RT-qPCR and sequencing, detecting DENV RNA, but were unable to resolve serotypes due to short sequencing fragments [22]. Wolfe et al. (Miami-Dade, Florida) successfully detected DENV RNA in solid fractions using RT-ddPCR [24], whereas magnetic bead-based concentration followed by RT-qPCR in Guangzhou, China resulted in limited detection consistent with low local transmission [20]. Conversely, Thakali et al. (Nepal) employed an electronegative membrane–vortex concentration method coupled with RT-qPCR and RT-dPCR but achieved no detection (Table 2 and Table 3) [23]. Beyond quantitative detection, sequencing was applied to provide molecular epidemiological context. Ma et al. used sequencing to identify circulating DENV lineages and validate wastewater signals against matched clinical samples [20], Araújo et al. applied hybrid-capture sequencing for broad multi-virus screening and confirmation of PCR detections [19], and Monteiro et al. used amplicon sequencing to confirm DENV or chikungunya virus (CHIKV) detections and provide phylogenetic context [22]. Collectively, these studies indicate that while DENV detection in wastewater remains inconsistent across regions and outbreak intensities, approaches that integrate solid fraction analysis with advanced molecular techniques particularly RT-dPCR offer higher sensitivity, specificity, and reliability for wastewater-based dengue surveillance.

### 3.4. Detection of DENV Through Spiked Study

The four included studies showed wide variation in substantial methodological heterogeneity in clarification, concentration workflows, membrane materials, processed volumes, and analytical platforms. Serotype coverage also varied, one study detected all four DENV serotypes [27], one study only targeted DENV1 [28], and two studies focused on DENV2 and DENV3 [25,26]. Before concentration, wastewater is often clarified to remove suspended solids that can clog equipment or interfere with assays. Study by Roldan-Hernandez et al. determined that filtration through a 0.22 μm filter is superior to centrifugation (4000× *g* for 1 h), resulting in significantly lower viral loss (approximately 9.06% compared to much higher losses for some viruses during centrifugation). Solid–Liquid Partitioning, alternatively, centrifugation (24,000× *g* for 30 min) is used specifically to separate solid and liquid fractions to study viral partitioning. This research highlights that, pre-clarification by filtration minimizes viral loss, while high-speed centrifugation enables solid–liquid partitioning, revealing markedly higher viral enrichment in solids [28].

As for the sample concentration part, centrifugal ultrafiltration achieved the highest recoveries, outperforming polyethylene glycol (PEG) precipitation and membrane capture [25]. RNA extraction with silica column kits (e.g., QIAamp Viral RNA Mini) showed robust yields. However, ultrafiltration performance varied substantially by device configuration and membrane material, not solely by molecular weight cut-off (MWCO). In the comparative evaluation by Chandra et al., centrifugal ultrafiltration was conducted using five single-use devices: Amicon^®^ 10 kDa (CU1), Amicon^®^ 30 kDa (CU2), Vivaspin^®^ 30 kDa (CU3), Spin-X^®^ 30 kDa (CU4), and Macrosep Advance^®^ 30 kDa (CU5). Among these devices, the 30 kDa centrifugal ultrafiltration unit with regenerated cellulose membrane (Macrosep Advance^®^, CU5) achieved the highest mean recovery (84.8%), with serotype-specific recoveries of 79.2% for DENV2 and 62.5% for DENV3. In contrast, alternative centrifugal devices with identical nominal MWCOs but different membrane chemistries and geometries (e.g., Amicon^®^, Vivaspin^®^, Spin-X^®^) yielded lower and more variable recoveries, demonstrating that recovery efficiency is strongly device- and material-dependent rather than determined by MWCO alone (Table 2) [24,25]. All studies used PCR-based detection with three studies using RT-qPCR [23,24,25], and one using RT-ddPCR [28]. These findings indicate that both RT-qPCR and RT-ddPCR effectively detect spiked DENV in wastewater, with RT-ddPCR offering superior sensitivity at low copy numbers. Process and extraction controls were included to assess recovery and extraction efficiency in all of the studies.

### 3.5. Quality Control and Detection Thresholds

Across dengue wastewater-based studies, methodological approaches varied in both analytical LOD or LOQ, quality control and the treatment of non-detects. LOD or LOQ values were reported using heterogeneous metrics across studies, whereas LOQ was rarely specified, with several studies relying on qualitative amplification thresholds to define positivity. Digital PCR–based studies applied minimum droplet or partition thresholds to ensure data quality, with wells containing fewer than 10,000 droplets excluded in droplet-based platforms [28], and nanoplate-based systems reporting high proportions of valid partitions per reaction ranging from 23,199 to 26,000 [23]. Criteria for defining positive detections also differed, ranging from minimum positive droplet counts of at least three droplets [24] to statistically derived limits of detection corresponding to 3.995 genome copies per reaction [21]. Approaches for handling non-detects were heterogeneous: some studies applied imputation using a fraction or the full value of the LOD or substitution of non-detects with LOD [22,28], others excluded non-detects from quantitative summaries [22], while several reported results qualitatively as below detection limits when viral RNA was not detected [23,24]. These variations highlight the lack of standardized practices for data handling and reporting in dengue WBE.

### 3.6. Risk of Bias Assessment

**Figure 2:**
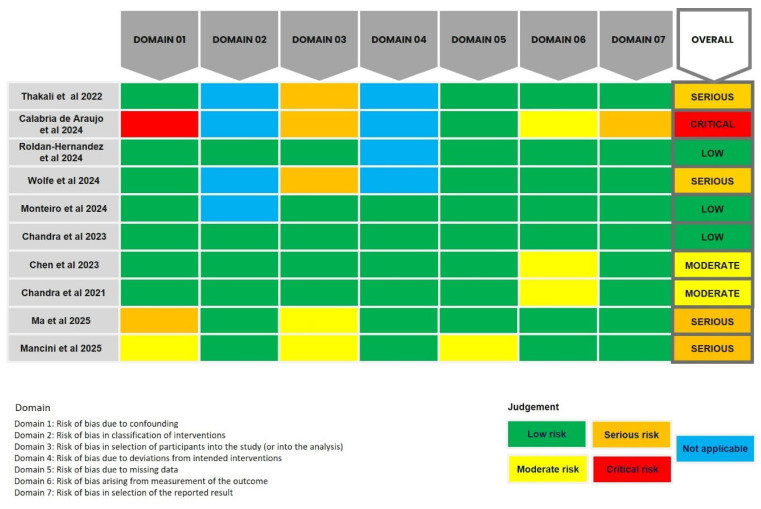
Risk of bias assessment of the included studies across seven domains [19,20,21,22,23,24,25,26,27,28]. Risk judgements are shown as low risk (green), moderate risk (yellow), serious risk (orange), critical risk (red), and not applicable (blue). Overall risk assessments are summarized in the rightmost column.

Risk of bias (RoB) was assessed across seven domains using ROBINS-I, with a summary presented in Figure 2 and detailed outcome-level judgements reported in Supplementary Table S4. Domains related to interventions (Risk of bias in classification of interventions: Domain 2 and Risk of bias due to deviations from intended interventions: Domain 4) were deemed not applicable for some observational study designs. Three studies were assessed as having an overall low risk of bias [22,25,28], while two studies had moderate risks [26,27]. Four studies were rated as having a serious overall risk of bias, mainly related to bias due to confounding (Domain 1) and sample selection (Domain 3) [20,21,23,24]. In particular, samples collected at WWTPs involved longer travel distances and times from the source, which may result in viral RNA degradation and dilution, thereby increasing the risk of bias estimates. Notably, one study was judged to have a critical risk of bias due to the absence of information on normalization techniques to control confounding factors, as well as serious risks related to sample selection and selective result reporting (Supplementary Table S4) [19].

The RoB assessment was conducted to inform the interpretation of results and were not used to determine study exclusion. All eligible studies were retained in the synthesis to reflect the limited evidence base. However, RoB was used to guide interpretation, including pooled estimates, with findings from studies with serious or critical risk of bias interpreted with caution. Studies with potential confounding or selection bias were considered likely to contribute to heterogeneity and may bias overall estimates. Differences across results were discussed in view of these methodological limitations identified through ROBINS-I assessment.

### 3.7. Meta-Analysis Outcomes

**Figure 3:**
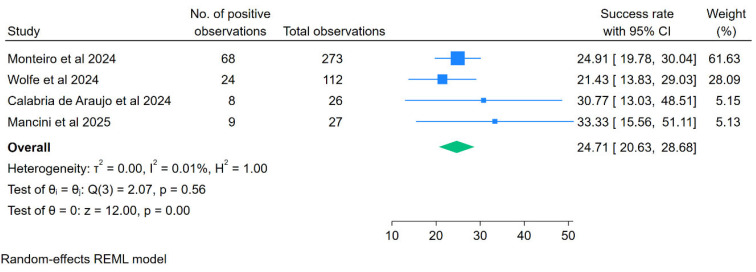
Forest plot of the meta-analysis after conducting sensitivity analysis, including studies [19,21,22,24].

A total of six articles were identified as being relevant to the purpose of the present meta-analysis. The heterogeneity was high, with an I^2^ value of 96.18%. Subsequently, sensitivity analysis was conducted using the leave-one-out method, generating an I^2^ value of 0% after omitting two studies [20,23]. The omitted studies suffered from compromised sample integrity and sensitivity thresholds that were not met due to specific environmental conditions (low prevalence [20]; retrospective risk samples analysis [23]). Including these studies would introduce significant heterogeneity (I^2^) because their negative or near-negative results stem from methodological limitations (storage, extraction method, sampling location) rather than a true absence of the virus or the general failure of WBE technology under optimal conditions. Hence, the final meta-analysis yielded a positivity rate of 24.71% (95% CI: 20.63–28.68%) as shown in Figure 3 below. As only 4 studies were included in the meta-analysis, the funnel plot to detect publication bias was not feasible. Furthermore, subgroup analysis according to sampling site types and detection methods was not applicable in this study.

## 4. Discussion

### 4.1. Overview of DENV Detection Through WBE

The use of WBE in disease surveillance is particularly relevant for arboviruses, given the high degree of underreporting associated with these infections. Global burden estimates indicate that asymptomatic dengue infections substantially outnumber symptomatic cases. In 2010 alone, approximately 293.9 million asymptomatic infections occurred worldwide compared with 96 million symptomatic infections, suggesting that nearly three-quarters of infections may go clinically unrecognized [1]. In addition, laboratory-confirmed diagnoses may not fully capture real-time community transmission dynamics, as confirmation and reporting processes can lag behind ongoing viral circulation [1,29]. Studies in various regions have provided evidence that DENV RNA can be detected in wastewater, highlighting its potential as a complementary surveillance tool. Nonetheless, the observed positivity rates differ markedly, reflecting differences in outbreak intensity, sampling methods, and methodological choices. Reported detection rates ranged from 33.3% during the DENV-2 outbreak in Central Italy [21] to only 2.3% detection of DENV-1 in Guangzhou, China, during community-level monitoring [20]. This variability emphasizes that while WBE offers potential for dengue surveillance, consistent detection remains technically challenging.

### 4.2. Methodological Determinants of Detection Success

The performance of DEN WBS is mostly affected by methodological variability, such as sample type, concentration approach, and molecular detection method. The stability and behavior of DENV which is an enveloped virus necessitates the use of optimized concentration and extraction techniques. Virus-associated RNA, predominantly contained within intact or partially degraded virions and virus–particle complexes, preferentially partitions to wastewater solids, leading to higher recoveries from the solid fraction compared with the liquid phase [21,24,28]. Enrichment in solids is often several orders of magnitude higher than in liquids, with reported partitioning coefficients of 10^3^–10^4^ mL g^−1^ [28]. Accordingly, positive detections frequently occur predominantly or exclusively in the solid fraction, whereas paired liquid samples often yield no detectable signal [21,24]. This preferential recovery from solids is consistent with adsorption of viral particles and RNA fragments onto particulate matter, a process that leads to viral concentrations several orders of magnitude higher in wastewater solids than in the liquid phase for many viruses and enhances analytical sensitivity for WBE [21,30,31]. Various concentration methods including electronegative membrane filtration, magnetic bead capture and ultrafiltration produce significantly different sensitivities. Magnetic bead–based and hollow-fiber ultrafiltration methods exhibit strong recovery efficiency and adaptability to large volumes, whereas low-volume filtration methods often show reduced efficiency when viral loads are minimal or RNA degradation is high.

### 4.3. Key Findings

#### 4.3.1. Global Trends and Variability in Detection Rates

The heterogeneity in wastewater detection reported across studies underscores the close relationship between DENV signal strength and underlying epidemiological context. Higher detection rates are typically observed during defined outbreaks, when viral shedding into sewer systems peaks [21]. Conversely, areas with low or nascent dengue activity illustrate how wastewater signals naturally decline when population infection levels are lower, such as Guangzhou, China, and Nepal, demonstrating low or absent wastewater signals despite active ongoing clinical surveillance [23,24]. These patterns highlight the importance of aligning WBE sampling strategies with epidemiological peaks to maximize detection reliability. Seasonal trends observed in Europe further demonstrate that wastewater signals can track temporal shifts in dengue activity [22]. Overall, WBE effectiveness is governed by multiple interrelated factors, including viral shedding dynamics, environmental RNA persistence, and the representativeness of collected wastewater samples, factors that are often region- and outbreak-specific. A thorough understanding of how these parameters interact across different settings is vital for optimizing WBE frameworks and strengthening early warning and outbreak response [32,33].

#### 4.3.2. Serotype Identification and Genomic Insights

Across the three studies that explicitly applied sequencing in this review, wastewater-based sequencing complemented PCR-based detection by providing genetic validation, lineage resolution, and broader pathogen context [19,20,22]. In Guangzhou, whole-genome and Sanger sequencing confirmed wastewater signals against matched serum/urine and resolved circulating DENV1 genotypes (I and III), revealing cryptic transmission preceding clinical diagnosis and highlighting greater RNA degradation in wastewater than clinical matrices [20]. In Brazil, hybrid-capture whole-genome sequencing profiled the sewage virome, detecting Mpox alongside multiple viral families and corroborating RT-qPCR findings, thereby supporting early identification of emerging pathogens at community and hospital scales [19]. In Portugal, amplicon sequencing validated DENV/CHIKV detections via high homology to reference sequences and provided phylogenetic context in a non-endemic setting, informing potential importation pathways [22]. Collectively, these studies indicate that sequencing improves specificity and supports molecular epidemiology but is constrained by RNA degradation, limited sequencing depth, and cost. As a result, serotype resolution remains challenging in WBE, particularly when RNA is scarce or fragmented; for example, DENV RNA was detected in Portugal without serotype assignment due to short sequencing reads [21]. Serotype identification is feasible when RNA integrity and sequencing depth are sufficient. Given the low shedding of arboviruses relative to enteric viruses, achieving adequate sensitivity often requires processing larger wastewater volumes (typically ≥ 1–5 L), as reported for DENV and ZIKV [15].

#### 4.3.3. Integration of Clinical and Vector Surveillance

Most included studies did not operationalize wastewater data within formal public health decision-making frameworks. However, they provide important methodological insights. These proposed integration pathways represent future directions rather than practices currently validated by the reviewed literature. The potential of WBE extends beyond simple detection; it provides a near-real-time reflection of population-level infection dynamics that can be operationalized for public health action. When integrated with clinical notifications and entomological indicators, wastewater data can contribute to a multi-layered surveillance network in which predefined wastewater thresholds or sustained increases in viral RNA from specific sewersheds trigger pre-emptive intensification of Aedes control, targeted diagnostic testing, enhanced syndromic surveillance and tailored community messaging. The temporal alignment between wastewater viral trends and vector indices may contribute to early warning frameworks and outbreak preparedness when integrated with other surveillance systems [14].

At present, evidence supporting a consistent early warning advantage of dengue WBE over clinical or entomological surveillance remains limited. Existing studies are largely based on proof-of-concept or outbreak-specific investigations, with few demonstrating a reproducible lead-time advantage across different epidemiological settings. Furthermore, the low and variable levels of viral shedding, together with methodological heterogeneity, may affect the sensitivity and temporal reliability of wastewater signals. As such, dengue WBE is currently better positioned as a complementary surveillance approach rather than a standalone early warning system, pending further validation through longitudinal and multi-site studies.

In the United States, the co-detection of DENV3 RNA in wastewater with increasing clinical cases demonstrated that WBE may provide supportive or complementary signals alongside clinical surveillance [24]. Similar integration was achieved in Portugal, where wastewater detections showed temporal alignment with clinical case trends, suggesting potential earlier signals. When coupled with Aedes mosquito surveillance, WBE can inform geographically targeted interventions, optimize vector control resource allocation, and strengthen preparedness frameworks in both endemic and non-endemic regions.

#### 4.3.4. Meta-Analytic Interpretation and Limitation

Currently, there is no standardized “gold standard” method for DENV detection in wastewater, making a standardized detection probability impossible to calculate across studies. The “positivity rate” serves as the only common denominator to assess the feasibility of surveillance across vastly different methodological approaches. Our analysis reveals that “positivity” is largely a function of the specific matrix and sampling strategy used rather than just disease prevalence.

The pooled positivity rate of 24% (95% CI: 20–28%) demonstrates that DENV and other arboviruses can be detected through WBE, but within a narrow analytical window. Rather than reflecting random variability, the heterogeneity observed across studies reveals a consistent interplay between viral biology, transmission intensity, and methodological design. Because DENV shedding into wastewater is sparse and episodic with concentrations approximately 100-fold lower than Severe Acute Respiratory Syndrome Coronavirus 2 (SARS-CoV-2) the detectability of its RNA depends strongly on aligning sampling and concentration workflows with periods of heightened community transmission. Where incidence was low, grab samples, small volumes, and concentration techniques optimized for non-enveloped viruses collectively reduced the likelihood of capturing already weak signals. This is further fueled by low sample sizes (ranging between 8–68) and inability to create a funnel plot due to the low number of included studies for meta-analysis (n-4).

The exclusion of long-duration, low-prevalence datasets [20,23] reflects this methodological–biological coupling, as prolonged storage under suboptimal conditions (e.g., −25 °C) and low transmission levels likely suppressed detectable signals. Collectively, these findings indicate that improving arboviral WBE performance will require surveillance systems that explicitly account for the instability of enveloped RNA viruses, including the need for larger sample volumes, appropriate concentration strategies, and sampling designs that coincide with epidemiological peaks. By integrating biological constraints with methodological optimization, future wastewater surveillance frameworks can more reliably support improved surveillance and situational awareness of dengue and related arboviruses.

The robustness of meta-analysis is dependent on both sample size (power) and publication bias (reporting bias). Therefore, the low sample sizes of the four studies analyzed, ranging from 26 to 273 observations, do not produce large “study-effects”. Apart from that, conducting Egger’s Regression Test to determine its publication bias is not feasible due to the low number of studies (n = 4) which may cause the pooled effect to be heavily biased.

### 4.4. Study Limitation

DENV WBE is primarily limited by the low viral load in wastewater, estimated to be approximately two orders of magnitude lower than that of SARS-CoV-2 [20,34,35]. DENV’s enveloped, single-stranded RNA structure renders it highly susceptible to degradation, particularly under tropical conditions or prolonged storage [36,37]. Environmental factors such as pH, temperature, and organic content further accelerate RNA decay, leading to reduced detection sensitivity [38,39]. Retrospective testing of archived wastewater samples, particularly for enveloped RNA viruses such as DENV, is highly vulnerable to signal loss and may lead to false-negative interpretations.

Moreover, the presence of PCR inhibitors, especially humic acids and surfactants common in tropical wastewater, can compromise amplification efficiency [40,41]. Studies have also reported inconsistencies between grab and composite sampling approaches, where temporal variability in viral shedding may lead to stochastic detection [42,43]. Thus, careful optimization of pre-analytical steps including rapid sample preservation, solid–liquid partitioning, and inhibitor removal is critical for reproducible results. Biologically, asymptomatic infections complicate the correlation between wastewater viral loads and reported case counts. In endemic regions, persistent low-level viral shedding may obscure emerging outbreak signals, while in non-endemic regions, sporadic detections can represent imported cases rather than local transmission. This emphasizes the need for context-specific calibration when interpreting wastewater data for DENV surveillance. DENV RNA detected in wastewater may arise from a combination of human excreta and environmental inputs, including vector-derived sources such as infected mosquitoes or larvae. The relative contribution of these sources remains poorly quantified, which introduces uncertainty in the interpretation of wastewater signals.

Despite growing evidence of feasibility, significant methodological gaps persist. Current concentration methods show variable recovery rates, and viral partitioning between solid and liquid fractions remains poorly understood. Further comparative studies are needed to evaluate the efficiency of emerging concentration technologies, such as ultrafiltration cartridges and graphene oxide–based adsorbents, for enveloped viruses like DENV.

### 4.5. Recommendation and Future Directions

#### 4.5.1. Innovations and Future Directions in Dengue WBE

Future research should also focus on establishing robust quality control standards, including recovery efficiency benchmarks and process control viruses. Advances in high-sensitivity quantification tools such as RT-dPCR and Clustered Regularly Interspaced Short Palindromic Repeats (CRISPR) based detection could improve analytical resolution and facilitate low-copy detection in small sample volumes. Integration with portable sequencing platforms, like nanopore sequencing, may further enhance rapid serotype identification and outbreak source tracking. Moreover, machine learning–driven modelling approaches can help calibrate WBE signals against epidemiological data, accounting for confounders such as rainfall, wastewater dilution, and population mobility. Developing predictive models that integrate WBE, meteorological, and vector surveillance data could support more proactive dengue management approaches.

#### 4.5.2. Policy Implications and Surveillance Equity

Implementing DENV WBE at scale will therefore require careful attention to laboratory and operational feasibility, especially in resource-limited settings. A phased approach focusing first on a small number of strategically selected sewersheds, prioritizing analysis of solids and the use of RT-dPCR where feasible and integrating workflows into existing public health laboratories may offer a pragmatic pathway for gradual scale-up. In lower-capacity contexts, regional or reference laboratories, shared protocols and external quality assessment schemes can help ensure data comparability while avoiding duplication of effort.

From a public health perspective, implementing DENV WBE requires not only technical optimization but also institutional support and sustainable investment. The early success of SARS-CoV-2 WBE networks offers a practical model for integrating arbovirus monitoring into existing infrastructure. However, low- and middle-income countries often at the epicenter of dengue transmission face challenges including limited laboratory capacity, resource constraints, and inconsistent data reporting mechanisms.

To ensure equitable access, international partnerships and funding programs must prioritize technology transfer and capacity building. Establishing regional reference laboratories, standard data repositories, and joint training initiatives can help create a more inclusive global WBE framework. Policymakers should also consider embedding WBE outputs into existing public health decision-making processes, linking wastewater signals with vector control responses and community health communication strategies.

#### 4.5.3. Broader Applications and Comparative Insights from Other Pathogens

WBE’s application to DENV is part of a broader shift toward genomic environmental surveillance across multiple pathogens. Its success in monitoring SARS-CoV-2, poliovirus, and mpox virus demonstrates the feasibility of population-scale genomic monitoring systems [19,44,45]. The expansion of hybrid-capture sequencing and metagenomic pipelines has enabled more precise detection of viral variants in complex environmental matrices, even at low concentrations [46,47]. Nevertheless, translating these successes to DENV surveillance requires tailored methodological frameworks due to DENV’s inherent instability and low wastewater concentrations. Lessons from SARS-CoV-2 WBE indicate that standardized sampling volumes, transport logistics, and quantification controls are essential for inter-laboratory comparability [48]. Therefore, developing similar standardized operating procedures for arboviral WBE is crucial for data harmonization and for enabling international data sharing during outbreak events.

## 5. Conclusions

The present synthesis highlights that DENV can be detected in wastewater across a range of settings, but achieving consistent results remains challenging. Detection success is strongly influenced by viral concentration, serotype, environmental persistence, and methodological rigor. High-sensitivity techniques such as RT-dPCR and sequencing-based confirmation provide the most reliable outcomes, particularly when applied to wastewater solids. For dengue control programs, these findings suggest that implementing WBE is most likely to be informative when focusing on solids, using RT-dPCR, and embedding wastewater data into existing clinical and entomological surveillance systems rather than treating it as a stand-alone tool. In this context, WBE can capture both symptomatic and asymptomatic infections and support improved surveillance and situational awareness. As methodological standardization advances and further validation studies are conducted across diverse settings, wastewater monitoring has the potential to contribute to integrated dengue surveillance frameworks.

## Figures and Tables

**Table 1:** Overview of study characteristics and sample collection.

Study Characteristics						Sample Collection		
Authors	Country	Sampling Site	Population Served	Sample Type	Sampling Approach	Sampling Frequency	Sampling Duration	Storage Conditions
Surveillance study								
Calabria De Araujo et al., 2024 [19]	Brazil	WWTP, Hospitals sewer	More than 1,000,001	Raw sewage (influents, untreated)	Composite	Bimonthly	9 months (July 2022–March 2023)	Viral concentrates were stored at −80 °C
Ma et al., 2025 [20]	China	Manhole, WWTP	Not explicitly mentioned	Raw sewage (influents, untreated)	Grab Composite	Daily	5–9 days (May 2024)	Storage for transportation: at 4 °C
Mancini et al., 2025 [21]	Italy	WWTP	15,000 to 116,500	Raw sewage (influents, untreated), Wastewater solid	Composite	Twice per week	1 month (1 and 30 October)	N/A
Monteiro et al., 2024 [22]	Portugal	WWTP	100,001 to 1,000,000	Raw sewage (influents, untreated)	Composite	Bimonthly	11 months (16 May 2022–19 April 2023)	Storage for transportation: 4 °C within 8-h window Concentrated samples were stored at −80 °C (±10) until further processing (e.g., extraction)
Thakali et al., 2022 [23]	Nepal	WWTP, Hospitals sewer, river	more than 1,000,001	Raw sewage (influents, untreated), Effluents (treated), aeration tank	Grab	Not explicitly mentioned	3 years (2017–2019)	Viral concentrates were stored at −25 °C for a long period of time (two to three years) until RNA extraction in September 2021
Wolfe et al., 2024 [24]	United States	WWTP	More than 1,000,001	Wastewater solid	Composite	Three times per week	3 months (June–Sept 2023)	Storage for transportation: at 4 °C
Experimental study								
Chandra et al., 2023 [25]	Singapore	Manholes (college residential buildings)	100 to 1000	Raw sewage (influents, untreated)	Composite	From 3 am to 3 am next day	1 day (24-h)	Storage for transportation: at 4 °C Extracted RNAs were stored at −80 °C less than a month prior to RT-qPCR
Chandra et al., 2021 [26]	Singapore	Office building (maintenance hole that collates sanitary lines)	Not explicitly mentioned	Raw sewage (influents, untreated)	Composite	8 h (9 am–5 pm) aggregate samples	Not explicitly mentioned	During collection and transport, the wastewater was collected into a polypropylene bottle on ice (at 4 °C) All RNA samples were stored at −80 °C prior to RT-qPCR Propagated viral stocks snap-frozen in liquid nitrogen in the presence of 20% FBS and 10% (*w*/*v*) sorbitol Non-cryopreserved DENV-2 samples were incubated at 25 °C
Chen et al., 2023 [27]	Taiwan	WWTP, University Hospital	Not explicitly mentioned	Raw sewage (influents, untreated)	Not explicitly mentioned	Not explicitly mentioned	Not explicitly mentioned	Maintained at 4 °C during transport to the laboratory
Roldan-Hernandez et al., 2025 [28]	United States	WWTP (primary sludge line)	More than 1,000,001	Wastewater	Not explicitly mentioned	Once at every location (weekly to biweekly)	5 months (October– December 2023)	Storage for transportation: at 4 °C Raw samples stored before spiking

FBS: fetal bovine serum, WWTP: wastewater treatment plant, RT-qPCR: quantitative reverse transcription polymerase chain reaction, RNA: ribonucleic acids, N/A: not applicable.

**Table 2:** Laboratory and analytical analysis.

Author (Year)	Concentration Method and Initial Volume for Concentration	Detection Method	* Normalization Performed	Process Control	Sequencing Performed	Dengue Serotypes Identified
Surveillance study						
Calabria De Araujo et al. 2024 [19]	Electronegative membranes (30 to 50 mL)	RT-qPCR	Human Adenovirus, JC Polyomavirus	Not explicitly mentioned	Whole genome sequencing (WGS) hybrid capture	DENV1
Ma et al. 2025 [20]	Polyethylene Glycol (PEG) Precipitation (50 mL) Magnetic Bead-Based (15 mL)	RT-qPCR,	Not explicitly mentioned	PMMoV (Recovery Efficiency), murine hepatitis virus (MHV) (Sample Processing Control)	Sanger Sequencing	Only DENV1 detected, non-detect for DENV2, DENV3, and DENV4 RNA
Mancini et al. 2025 [21]	PEG/ sodium chloride precipitation (40 mL), Nanotrap^®^ Magnetic Virus Particles (40 mL), Electropositive membrane filtration (1 L).	RT-qPCR, RT-dPCR	Not explicitly mentioned	Mengovirus (Recovery Efficiency),	Attempted nested PCR for sequencing	DENV2
Monteiro et al. 2024 [22]	Hollow fibre (Innovaprep Concentrating Pipette) (80 mL)	RT-qPCR	crAssphage DNA	PEDV, Murine norovirus (extraction control)	Amplicon sequencing	DENV
Thakali et al. 2022 [23]	Electronegative membrane- vortex (EMV) (50 mL)	RT-qPCR, RT-dPCR	PMMoV	Pseudomonas bacteriophage	Not explicitly mentioned	Not detected
Wolfe et al. 2024 [24]	Centrifugation (50 mL)	RT-ddPCR	PMMoV	BCoV	Not explicitly mentioned	Only DENV3 detected, non-detect for DENV1, DENV2, and DENV4 RNA
Experimental Study						
Chandra et al. 2023 [25]	Centrifugal ultrafiltration, PEG precipitation, Charged Membrane Capture and Elution, Hollow-Fiber Ultrafiltration (80 mL)	RT-qPCR	Not explicitly mentioned	MHV	Not explicitly mentioned	Spiked DENV2 and DENV3
Chandra et al. 2021 [26]	Gentle centrifugation at 525 g (40–200 mL)	RT-qPCR	Not explicitly mentioned	PPMoV (extraction control)	Not explicitly mentioned	Spiked DENV2 and DENV3
Chen et al. 2023 [27]	Ultrafiltration, Flocculation with skim milk (50 mL)	RT-qPCR	Not explicitly mentioned	HCV, JFH-1	Not explicitly mentioned	Spiked DENV1–4
Roldan-Hernandez et al. 2025 [28]	Centrifugation (50 mL)	RT-ddPCR	Not explicitly mentioned	BCoV	Not explicitly mentioned	Spiked DENV1

RT-qPCR: reverse transcription quantitative polymerase chain reaction, RT-ddPCR: reverse transcription droplet digital PCR, PMMoV: pepper mild mottle virus, crAssphage: CrAss-like phages, DNA: deoxyribonucleic acid, DENV: dengue virus, PEDV: porcine epidemic diarrhea virus, BCoV: bovine coronavirus, DENV1–4: dengue virus serotype 1–4, JC polyomavirus: John Cunningham, MHV: murine hepatitis virus, HCV: hepatitis C virus, JFH-1: specific strain of the HCV, N/A: not applicable. * The approach to normalization and process controls of experimental studies differs from typical population-based wastewater surveillance studies that track ambient viral loads.

**Table 3:** Outcome and the epidemiological links.

Author (Year)	Sample Positivity (%)	Prevalence in Area	* Outcome Measure	Viral Load/Recovery Efficiency	Correlation with Epidemiological Data	Aims of the Study
Surveillance study						
Calabria De Araujo et al., 2024 [19]	8/26 hospital sewage samples (30.8%) 6/30 samples from municipal WWTPs A and B (20.0%)	Not explicitly mentioned	N/A	Not provided	Yes	Investigate the circulation of Mpox virus and other human viruses in sewage samples collected from municipal and hospital wastewaters in Belo Horizonte, Brazil
Ma et al., 2025 [20]	14/618 samples from manhole (2.3%) below detection limits for WWTP	Low-prevalence area experiencing an active outbreak	Viral load (copies number), Recovery efficiency	DENV1 RNA concentrations in urine samples ranged from 0.007 to 50.07 cp/L Concentration: (i) Magnetic bead: 59.7% (ii) PEG: 50.3% LOD: 10 cp/mL	Yes	Implement and test a community-level WBE system for dengue during an outbreak, assessing its ability to detect hidden transmission and guide public health action
Mancini et al., 2025 [21]	9/27 samples from WWTP (33.3%)	Outbreak and non-outbreak area	Viral load concentrations (genome cp/g), quantification cycle (Cq) values, and percentage recovery efficiency	DENV2: 6.1 × 10^1^ to 7.9 × 10^2^ cp/gram of solid material Ct value: 41.41 to 43.38 PEG/NaCl precipitation: 43.4% Nanotrap^®^ Magnetic Virus Particles (Ceres Nanosciences, Inc., Manassas, VA, USA): 6.1% Electropositive membrane filtration:3.0% LOQ: lowest standard able to be quantified with CV value below 35%	Yes	Investigate the feasibility of using WBE to detect dengue virus serotype 2 RNA during a local outbreak
Monteiro et al., 2024 [22]	68/273 (25.0%)	Low prevalence area (low cases area/ non- endemic area)	Viral load (copies number)	Median normalized 1.1 × 10^−4^ IQR 3.2 × 10^−5^ to 8.0 × 10^−4^) Non-normalized median DENV RNA concentration was 8.6 × 10^4^ cp/L (IQR 3.1 × 10^4^ to 4.5 × 10^5^) LOD: 2.1 × 10^3^ cp/L	Yes, but only for 2021	Explore WBE as an additional tool for tracking DENV and CHIKV Assess the capability of WBE to enhance conventional clinical and vector surveillance Gain an understanding about the distribution and patterns of both DENV and CHIKV in wastewater in Portugal
Thakali et al., 2022 [23]	0/34 (0%)	low prevalence area (low cases area)	N/A	Not provided LOD: 4.2 log 10 cp/L using PCR and RT-dPCR	Yes	Investigate the presence of DENV RNA in the wastewater of the Kathmandu Valley, Nepal
Wolfe et al., 2024 [24]	24 of 112 (21%)	Non-endemic area of the United States commonly experiencing both travel-associated and locally acquired DENV infections, and it regularly records the highest numbers of dengue cases in the United States outside of non-main land US territories	Copies per gram of dry weight (cp/g) of wastewater solids	Below the LOD of (~500 cp /g dry weight) to a maximum of 4.1 × 10^3^ cp/g (with a median of non-detect)	Yes	Demonstrate the feasibility and utility of wastewater monitoring for the detection of dengue virus RNA in a community
Experimental study						
Chandra et al., 2023 [25]	N/A	Not explicitly mentioned	Percentage of recovery efficiency	Sample Clarification (i) Filtration DENV2: 4.0% DENV3 12.2%, (ii) Centrifugation DENV2:38.5% DENV3:47.5% (iii) Filtration centrifugation Concentration (i) CU DENV2: CU5 > CU4 > CU2 > CU3 > CU1 (79.3 > 55.1 > 53.6 > 53.0 > 44.6) DENV3: CU5 > CU3 > CU2 > CU4 > CU1 (62.5 > 45.3 > 44.1 > 42.5 > 32.7) (ii) PEG DENV2: PEG1 = PEG2 (22.5, 22.8) DENV3: PEG1 > PEG2 (17.8 > 14.0) (iii) CM DENV2: CM1 > CM2 > CM3 (34.4 > 28.0 > 3.4) DENV3: CM1 > CM2 > CM3 (22.3 > 12.5 > 0.8) (iv) HF DENV2: HF1 = HF2 (3.0, 3.2) DENV3: HF2 > HF1 (4.0 > 2.2) CU showed the highest overall arbovirus recovery efficiency among the methods tested Extraction (EX2) and (EX4) showed significantly higher extraction efficiency compared to the (EX1). LOD: 1.42 ± 0.23 to 7.71 ± 1.35 GC/μL	N/A	Evaluating various methodologies for sample clarification, concentration, and nucleic acid extraction to optimize the detection and recovery of introduced arboviral including dengue and MHV RNA signals within wastewater matrices
Chandra et al., 2021 [26]	N/A	Not explicitly mentioned	Percentage	N/A Analytical LOD: determined via RT-ddPCR and standard curve and range from 1.42 ± 0.23 to 7.71 ± 1.35 GC/μL	N/A	Investigate the persistence (decay kinetics) of representative arboviruses in an untreated wastewater matrix
Chen et al., 2023 [27]	N/A	Not explicitly mentioned	percentage of recovery efficiency	Concentration (i) Ultrafiltration DENV1: 34.4 ± 8.6% DENV2: 32.3 ± 16.3% DENV3: 21.2 ± 34.4% DENV4: 91.8 ± 70.6% (ii) SMF SMF yielded less than 1% recovery. LOD: reported in Plaque Forming Units (PFU) per mL for each serotype: ◦ DENV1: 9.89 PFU/mL. ◦ DENV2: 3.22 PFU/mL. ◦ DENV3: 2.96 PFU/mL. ◦ DENV4: 2.68 PFU/mL	N/A	Establishing a reliable and quantitative method for detecting DENV in wastewater.
Roldan-Hernandez et al., 2025 [28]	Not explicitly mentioned	N/A	(cp/mL) for the liquid fraction and cp/g of dry weight for the solid fraction	Liquid fraction (CL): DENV RNA concentrations ranged from 0.002–30.5 cp/L for Batch 1 and 0.002–1.7 cp/L Solid fraction (Cs): DENV RNA concentrations ranged from 5 × 10^3^–6 × 10^7^ cp/g for Batch 1 and 5 × 10^3^–2 × 10^7^ cp/g LOD - liquid fraction: 3.0 cp/mL - solid fraction: 2000 cp/g	N/A	Examine the solid–liquid partitioning behaviour of specific viruses in wastewater by determining the partition coefficient of these viruses in wastewater

* % recovery rates were determined by average recovery rate. N/A: not available, IQR: Interquartile range, cp: copies, CV: coefficient of variation, PEG: PEG precipitation, LOD: limit of detection, Ct: cycle threshold, LOQ: limit of quantification, RT-dPCR: reverse transcription digital polymerase chain reaction, CU: centrifugal ultrafiltration, CM: charged membrane capture, HF: hollow-fiber ultrafiltration, SMF: skimmed milk flocculation.

## Data Availability

The data supporting the findings of this study are available within the publicly available published studies and its Supplementary Materials. Additional extracted datasets are available from the corresponding author upon request.

## Supplementary Materials

The following supporting information can be downloaded at: https://www.mdpi.com/article/10.3390/v18050531/s1, Table S1: Prisma 2020 Checklist; Table S2: Key search syntax for Pubmed, Embase, Scopus, and Web of Science databases from inception to June 2025; Table S3: Extraction table; Table S4: Detailed justification for each risk of bias (RoB) Judgement; Table S5: Assessment of Risk of Bias according to ROBINS-I; Table S6: Hand Search—search strategy and process.

## Abbreviations

The following abbreviations are used in this manuscript:

WBE	Wastewater-based epidemiology
DENV	Dengue virus
RNA	Ribonucleic acid
RT-dPCR	reverse transcription digital polymerase chain reaction
RT-ddPCR	reverse transcription digital droplet polymerase chain reaction
SARS-CoV-2	severe acute respiratory syndrome coronavirus 2
ZIKV	Zika virus
JEV	Japanese encephalitis virus
PRISMA	Preferred Reporting Items for Systematic Reviews and Meta-Analyses
MESH	Medical Subject Heading
WOS	Web of Science
RoB	Risk of bias assessment
Ct-value	Cycle Threshold values
ROBINS-I	Risk of Bias in Non-randomized Studies of Interventions
CI	confidence intervals
WWTP	wastewater treatment plants
