# Supplementary material for: Methodological Approaches to Dengue Virus Detection in Wastewater: A Systematic Review and Meta-Analysis of Positivity Rate
Source: Viruses. 2026 Apr 30;18(5):531. doi: 10.3390/v18050531 (PMC13211638; doi:10.3390/v18050531)
Supplement: Supplementary file 1 [file viruses-18-00531-s001.zip › SUPPLEMENTARY S3_ Standardized Data Extraction Form Template (2).pdf]

Table S3: Extraction table

(i) Overview of study characteristics and sample collection

**Study characteristics**							**Sample collection**		
Paper no.	Year	country	Sampling site	Population served	Sample type	Sampling Approach	Sampling frequency	Sampling duration	Storage conditions

(ii) Laboratory and analytical analysis

**Paper no.**	**Concentration method and initial volume for concentration**	**Detection method**	**Normalization performed**	**Process control**	**Sequencing performed**	**Dengue serotypes identified**

(iii) Outcome and epidemiological links

Authors	Sample Positivity (%)	Prevalence in area	**outcome measure	Viral load / recovery efficiency	Correlation with epidemiological data	Aims of the study
