# Supplementary material for: Methodological Approaches to Dengue Virus Detection in Wastewater: A Systematic Review and Meta-Analysis of Positivity Rate
Source: Viruses. 2026 Apr 30;18(5):531. doi: 10.3390/v18050531 (PMC13211638; doi:10.3390/v18050531)
Supplement: Supplementary file 1 [file viruses-18-00531-s001.zip › SUPPLEMENTARY S2_ Keyword Search Strategy (1) (1).pdf]

Table S2. Key search syntax for Pubmed, Embase, Scopus, and Web of Science databases from inception to October 2025

ID	Key search syntax
**Pubmed database**	
#1	"Waste Water"[Title/Abstract] OR "Sewage"[Title/Abstract] OR "Wastewater"[Title/Abstract] OR "wastewater treatment plant*"[Title/Abstract] OR "WBE"[Title/Abstract] OR "wastewater-based"[Title/Abstract] OR "sludge"[Title/Abstract] OR "untreated wastewater"[Title/Abstract]
#2	"surveillance"[Title/Abstract] OR "monitoring"[Title/Abstract] OR "epidemiology"[Title/Abstract] OR "tracking"[Title/Abstract] OR "tracing"[Title/Abstract] OR "wastewater surveillance"[Title/Abstract] OR "wastewater-based surveillance"[Title/Abstract] OR "wastewater-based epidemiology"[Title/Abstract]
#3	"Dengue"[Title/Abstract] OR "Dengue virus RNA"[Title/Abstract] OR "Arthropod-Borne"[Title/Abstract] OR "Arbovirus"[Title/Abstract] OR "Dengue viruses"[Title/Abstract] OR "Human arboviral disease"[Title/Abstract] OR "DENV"[Title/Abstract]
#4	(("Waste Water"[Title/Abstract] OR "Sewage"[Title/Abstract] OR "Wastewater"[Title/Abstract] OR "wastewater treatment plant*"[Title/Abstract] OR "WBE"[Title/Abstract] OR "wastewater-based"[Title/Abstract] OR "sludge"[Title/Abstract] OR "untreated wastewater"[Title/Abstract]) AND ("surveillance" OR "monitoring" OR "epidemiology" OR "tracking" OR "tracing" OR "wastewater surveillance" OR "wastewater-based surveillance" OR "wastewater-based epidemiology")) AND ("Dengue"[Title/Abstract] OR "Dengue virus RNA"[Title/Abstract] OR "Arthropod-Borne"[Title/Abstract] OR "Arbovirus"[Title/Abstract] OR "Dengue viruses"[Title/Abstract] OR "Human arboviral disease"[Title/Abstract] OR "DENV"[Title/Abstract]) Filters: **English**
**Embase database**	
#1	'waste water':ab,ti OR 'sewage':ab,ti OR 'wastewater':ab,ti OR 'wastewater treatment plant*':ab,ti OR 'wbe':ab,ti OR 'wastewater-based':ab,ti OR 'sludge':ab,ti OR 'untreated wastewater':ab,ti
#2	'surveillance':ab,ti OR 'monitoring':ab,ti OR 'epidemiology':ab,ti OR 'tracking':ab,ti OR 'tracing':ab,ti OR 'wastewater surveillance':ab,ti OR 'wastewater-based surveillance':ab,ti OR 'wastewater-based epidemiology':ab,ti
#3	'dengue virus rna':ab,ti OR 'arthropod-borne':ab,ti OR 'arbovirus':ab,ti OR 'dengue viruses':ab,ti OR 'human arboviral disease':ab,ti OR 'dengue':ab,ti
#4	#1 AND #2 AND #3 AND [english]/lim
**Scopus database**	
#1	TITLE-ABS ("Waste Water" OR "Sewage" OR "Wastewater" OR "wastewater treatment plant*" OR "WBE" OR "wastewater-based" OR "sludge" OR "untreated wastewater") AND (LIMIT-TO (LANGUAGE , "English"))
#2	TITLE-ABS ("surveillance" OR "monitoring" OR "epidemiology" OR "tracking" OR "tracing" OR "waste water surveillance" OR "wastewater-based surveillance" OR "wastewater-based epidemiology") AND (LIMIT-TO (LANGUAGE , "English"))
#3	TITLE-ABS-KEY ("surveillance" OR "monitoring" OR "epidemiology" OR "tracking" OR "tracing" OR "waste water surveillance" OR "wastewater-based surveillance" OR "wastewater-based

	epidemiology" "Dengue" OR "Dengue virus RNA" OR "Arthropod-Borne" OR "Arbovirus" OR "Dengue viruses" OR "Human arboviral disease" OR "DENV") AND (LIMIT-TO (LANGUAGE , "English"))
#18	TITLE-ABS-KEY (("Waste Water" OR "Sewage" OR "Wastewater" OR "wastewater treatment plant*" OR "WBE" OR "wastewater-based" OR "sludge" OR "untreated wastewater") AND ("surveillance" OR "monitoring" OR "epidemiology" OR "tracking" OR "tracing" OR "wastewater surveillance" OR "wastewater-based surveillance" OR "wastewater-based epidemiology") AND ("surveillance" OR "monitoring" OR "epidemiology" OR "tracking" OR "tracing" OR "wastewater surveillance" OR "wastewater-based surveillance" OR "wastewater-based epidemiology" "Dengue" OR "Dengue virus RNA" OR "Arthropod-Borne" OR "Arbovirus" OR "Dengue viruses" OR "Human arboviral disease" OR "DENV")) AND (LIMIT-TO (DOCTYPE , "ar")) AND (LIMIT-TO (LANGUAGE , "English"))
**Web of Science Database**	
#1	AB=("Waste Water" OR "Sewage" OR "Wastewater" OR "wastewater treatment plant*" OR "WBE" OR “wastewater-based” OR “sludge” OR “untreated wastewater”)
#2	AB=(“surveillance” OR “monitoring” OR “epidemiology” OR “tracking” OR “tracing” OR “wastewater surveillance” OR “wastewater-based surveillance” OR “wastewater-based epidemiology”)
#3	AB=(“Dengue” OR “Dengue virus RNA” OR “Arthropod-Borne” OR “Arbovirus” OR “Dengue viruses” OR “Human arboviral disease” OR “DENV”)
#4	((AB=("Waste Water" OR "Sewage" OR "Wastewater" OR "wastewater treatment plant*" OR "WBE" OR “wastewater-based” OR “sludge” OR “untreated wastewater”)) AND AB=(“surveillance” OR “monitoring” OR “epidemiology” OR “tracking” OR “tracing” OR “wastewater surveillance” OR “wastewater-based surveillance” OR “wastewater-based epidemiology”)) AND AB=(“Dengue” OR “Dengue virus RNA” OR “Arthropod-Borne” OR “Arbovirus” OR “Dengue viruses” OR “Human arboviral disease” OR “DENV”)
