# Supplementary material for: Methodological Approaches to Dengue Virus Detection in Wastewater: A Systematic Review and Meta-Analysis of Positivity Rate
Source: Viruses. 2026 Apr 30;18(5):531. doi: 10.3390/v18050531 (PMC13211638; doi:10.3390/v18050531)
Supplement: Supplementary file 1 [file viruses-18-00531-s001.zip › SUPPLEMENTARY S4_ Detailed Justification for Each Risk of Bias (RoB) Judgment (2).pdf]

Table S4: Detailed Justification for Each Risk of Bias (RoB) Judgment

RoB in Non-Randomised Studies – of interventions (ROBINS-I) Tool for Dengue WBE

Intervention = sampling methodologies (sample type, sampling techniques, frequency, location), detection and serotype analysis.

Outcome of interest: Viral load (gene copies, CT-value), positivity rate

Confounding : Environmental factors: including temperature, rainfall, or dilution effects and chemical in wastewater systems, could have impacted the detectability of viral RNA (low risk)

**Confounding domain**	**Definitions by ROBINS-I**	**When applying to Environmental surveillance of dengue: reporting of viral load quantification or positivity rate**
Pre-intervention	**Risk of bias assessment is mainly distinct from assessments of randomised trials**	
Bias due to confounding (DOMAIN 1)	Baseline confounding occurs when one or more prognostic variables (factors that predict the outcome of interest) also predicts the intervention received at baseline<br><br>ROBINS-I can also address time-varying confounding, which occurs when individuals switch between the interventions being compared and when post-baseline prognostic factors affect the intervention received after baseline	Outcome of interest:<br>Viral load (gene copies, CT-value), positivity rate<br><br>**Confounding factors :** Environmental factors: including temperature, rainfall, or dilution effects and chemical in wastewater systems, could have impacted the detectability of viral RNA (low risk if confounding is controlled through normalization)<br><br>Thakali et al. (2022): Samples collected during the monsoon period - in which dengue cases were expected to be high. Environmental confounding factors were controlled using normalization biological indicators (PMMoV). PMMov was used to determine the presence of PCR inhibitors and those samples had those were removed from further analysis. **(LOW RISK)**

		Araujo et al. (2024): No information on performance of normalisation. The results may have inhibits that can affect the findings. **(CRITICAL RISK)**<br><br>Roldan-Hernandez et al. (2024: Samples transportation and storage conditions were standardized. Confounding were controlled with BCoV for internal process control to assess the validity of RNA extractions. Positive and negative controls for extraction and PCR were positive and negative respectively. **(LOW RISK)**<br><br>Wolfe et al. (2024): Confounding for environmental factors were controlled using normalization with biological indicators (PMMoV,crAssphage) with all negative control yielded negative results . The authors analysed the viral quantities based total sampling period, calendar month and by season **(LOW RISK)**<br><br>Monteiro et al. (2024): Sample collection and processing was collected by trained personnel, temperature was throughout the transportation, PPMoV was use to counter dilution effects, recovery efficiency and validation was performed. **(low risk)**<br><br>**Chandra et al. (2023)**: The confounding factor for temperature and physicochemical character such as pH has been standardised. Although the samples were taken from 3 maintenance manholes but the samples was pooled later. **(LOW RISK)**<br><br>**Chen at al. (2023)**:This is an experimental study, where it was conducted under a controlled environment such as using artificially loaded water samples, temperature was controlled, method was following the manufacturers instructions. The different qPCR efficiency limit the accuracy of the WBE but the error rate was determined by spike control material with titers and using non-cognate spike control (**LOW RISK**).

		Chandra et al. (2021): Experimental study all Confounding factors were controlled (**LOW RISK**)<br><br>Veneri et al. (2025): Normalizing the viral signal by the weight of the solid matrix (g) serves as a critical method of **normalization**, which inherently mitigates the effects of dilution and flow variability, especially since DENV exhibits an affinity for particulate matter. Because the authors used a normalization method appropriate for the detected matrix (copies/g solids), they partially controlled the important confounding factor of dilution/matrix variability, thereby reducing the risk compared to absolute quantification. However, limitation of the delayed sampling start (beginning during the epidemic's decline), which prevented the assessment of temporal correlation between wastewater viral concentrations and clinical case numbers during the outbreak's peak (**MODERATE RISK OF BIAS**)<br><br>Ma et al. (2025): The analysis does not indicate that the final DENV-1 viral load results were normalized using flow rate or human normalization markers (like PMMoV, which was used only for recovery efficiency calculation. No report on the negative control analysis **(SERIOUS RISK OF BIAS)**
Bias in selection of participants into the study (DOMAIN 3)	When exclusion of some eligible participants, or the initial follow-up time of some participants, or some outcome events is related to both intervention and outcome, there will be an association between interventions and outcome even if the effects of the interventions are identical<br><br>This form of selection bias is distinct from confounding—A specific example is bias	Samples taken at WWTPs located for the community for bigger coverage or metropolitan with lower concentration due to longer distance and decay rate of the sample – serious risks<br>Sample taken at upstream (example: manhole, septic tank, residential) nearer to the source for smaller population equivalent considered as ‘hot spots’ or moderate risk (have to start with at least moderate - as no information on question 3.1)<br><br>Thakali et al. (2022): Wastewater samples were collected from WWTPs and hospital from same place for 3 years. **(SERIOUS RISK)**

	due to the inclusion of prevalent users, rather than new users, of an intervention	Araujo et al. (2024):Wastewater samples were collected from WWTPs and hospital (metropolitan area) **(SERIOUS RISK)**<br><br>Roldan-Hernandez et al. (2024): Samples were taken at WWTPs. However, the viruses were spiked into the WW to form virus cocktails at the desired concentration and further tested for RNA extraction and quantification. As the selection of sites does not affect the result, the risk of bias is low **(LOW RISK)**<br><br>**Wolfe et al. (2024)**: The participants in this review is the selection of WWTPs where the wastewater samples are collected to proceed with methods for quantifying viral concentrations. Three WWTPs chosen have similar number of population equivalent served and located in the same state in adjacent counties – **(SERIOUS RISK)**<br><br>Monteiro et al. (2024):Because of the selection of the sites was not based on prevalence, the justification was based on the Portugal overall **(LOW RISK)**<br><br>Chandra et al. (2023):Because the 4 different intervention/method using the same pooled samples **(LOW RISK)**<br><br>Chen at al. (2023):All the methods/interventions was predetermined prior to the start of the experiment (**LOW RISK**)<br><br>Chandra et al. (2021):Assignment of intervention groups occurred before the starting the experiment (**LOW RISK**)<br><br>Veneri et al. (2025): Site selection (only two WWTPs) and delayed sampling could underrepresent true viral presence, but all inclusion decisions preceded outcome assessment and were applied consistently. Bias direction = toward under-detection. (**MODERATE RISK OF BIAS**)

		Ma et al (2025):Bias in the selection of samples is moderate because the surveillance focused exclusively on wastewater samples collected from manholes within a 200-m radius of 8 already reported clinical cases, meaning the analysis was conducted only in high-prevalence hot spots identified by traditional case detection **(MODERATE RISK OF BIAS**)
At intervention	**Risk of bias assessment is mainly distinct from assessments of randomised trials**	
Bias in classification of interventions (DOMAIN 2)	Bias introduced by either differential or non-differential misclassification of intervention status<br><br>Non-differential misclassification is unrelated to the outcome and will usually bias the estimated effect of intervention towards the null<br><br>Differential misclassification occurs when misclassification of intervention status is related to the outcome or the risk of the outcome, and is likely to lead to bias	If the study compare different methods for sampling, concentration, RNA detection – then go through page 21 – 24 (ROBINS-I)<br><br>Thakali et al. (2022): There is no classification of intervention since the study only assesses the viral load in the wastewater. we considered differential misclassification and risk of bias as not relevant **(Not applicable).**<br><br>Araujo et al. (2024): There is no classification of intervention since the study only assesses the viral load in the wastewater. we considered differential misclassification and risk of bias as not relevant **(Not applicable)**<br><br>Roldan-Hernandez et al. (2024): Wastewater was tested for concentrations of viral cocktails for liquid and solid WW samples. However, any measurement errors were likely non-differential as methods for sample collection and analysis were equal for all groups of virus studied. **(LOW RISK)**<br><br>Wolfe et al. (2024): There is no classification of intervention since the study only assesses the viral load in the wastewater. we considered differential misclassification and risk of bias as not relevant **(Not applicable)**

		Monteiro et al. (2024): There is no classification of intervention since the study only assesses the viral load in the wastewater. We considered differential misclassification and risk of bias as not relevant **(not applicable)**<br><br>Chandra et al. (2023): All methods were correctly assigned to the intended virus **(LOW RISK)**<br><br>Chen at al. (2023):All methods were correctly classified(**LOW RISK**)<br><br>Chandra et al. (2021): Intervention defined prior to the experiment (**LOW RISK**)<br><br>Veneri et al. (2025):The classification of interventions (different viral concentration and detection methods) was predefined and documented at the start of sampling, with no evidence that classification was influenced by outcomes (**LOW RISK OF BIAS**)<br><br>Ma et al (2025): The sampling method (grab sampling vs composite) and handling uniformly applied to all sites, as well as analysis comparing magnetic bead and polyethylene glycol (PEG) concentration methods for viral recovery efficiency so any reduction in sensitivity was non-differential across locations and unlikely to bias comparisons or overestimate detection**.** The effect is possible under-estimation of viral presence, but not distortion of the association between wastewater and clinical detections. Since the study aimed to evaluate feasibility and validity, not effect size, this limitation slightly weakens sensitivity but does not materially bias conclusions.(**LOW RISK OF BIAS**)
**Post-intervention**	**Risk of bias assessment has substantial overlap with assessments of randomised trials**	

Bias due to deviations from intended interventions (DOMAIN 4)	Bias that arises when there are systematic differences between experimental intervention and comparator groups in the care provided, which represent a deviation from the intended intervention(s)<br><br>Assessment of bias in this domain will depend on the type of effect of interest (either the effect of assignment to intervention or the effect of starting and adhering to intervention).	If there are changes in the method example for the first use grab, second year use composite, or changes in frequency of sampling, than go through page 29-32. If there are no changes in method, then this is not applicable<br><br>Thakali et al. (2022): The intervention in this study is the methods used for sampling which is grab method and analysis using RT-PCR to identify the number of copies for each DENV serotypes. The deviation from intended interventions is not applicable for this study **(Not applicable).**<br><br>Araujo et al. (2024): The intervention in this study is the methods used for sampling which is composite method and analysis whole genome sequencing. The deviation from intended interventions is not applicable for this study.**(Not applicable)**<br><br>Roldan-Hernandez et al. (2024): The deviation from intended interventions is not applicable for this study **(NOT APPLICABLE)**<br><br>Wolfe et al. (2024):The intervention in this study is the methods used for sampling which is grab method and analysis using RT-PCR to identify the number of copies for each DENV serotypes. The deviation from intended interventions is not applicable for this study **(NOT APPLICABLE)**<br><br>Monteiro et al. (2024):Low risk because there is no deviation between method **(LOW RISK)**<br><br>Chandra et al. (2023): There is no deviations for all the methods and analysis **(LOW RISK)**

		Chen at al. (2023): Study undertaken in experimental context with no deviation (**LOW RISK**)<br><br>Chandra et al. (2021):There is no deviations (**LOW RISK**)<br>Veneri et al. (2025): All samples underwent the same controlled laboratory procedures; deviations (e.g., inhibition removal steps) were corrective, not differential (**LOW RISK OF BIAS**)<br><br>Ma et al. (2025): No deviation from intended intervention. All sampling and laboratory procedures followed a consistent, standardized protocol. Minor procedural constraints, such as the use of grab sampling instead of 24-hour composites, reflect real-world operational practice rather than deviations due to the study context. These factors may reduce detection sensitivity uniformly but unlikely to introduce systematic bias or affect internal validity.(**LOW RISK**)

Bias due to missing data (DOMAIN 5)	Bias that arises when later follow-up is missing for individuals initially included and followed (such as differential loss to follow-up that is affected by prognostic factors); bias due to exclusion of individuals with missing information about intervention status or other variables such as confounders	Missing data is considered when sample are not collected on certain dates resulting in missing data for that particular period (go through page 35-40)<br><br>Thakali et al. (2022): All samples were taken during the allocated period. Results for the interventions (methods) used in this study are complete for all WWTP and hospitals with no missing data **(LOW RISK)**<br><br>Araujo et al. (2024): All samples were taken during the allocated period. Results for the interventions (methods) used in this study are complete for all WWTP and hospitals with no missing data **(LOW RISK)**<br><br>Roldan-Hernandez et al. (2024): All samples were taken during the allocated period. Results for the interventions (methods) used in this study is complete for all three WWTP with no missing data **(LOW RISK)**<br><br>Wolfe et al. (2024): All samples were taken during the allocated period. Results for the interventions (methods) used in this study is complete for all three WWTP with no missing data **(LOW RISK)**<br><br>Monteiro et al. (2024): No missing data**(Low risk)**<br><br>Chandra et al. (2023): no missing data **(LOW RISK)**<br><br>Chen at al. (2023): No missing data for each of the intervention(**LOW RISK**)<br><br>Chandra et al. (2021): No missing data (**LOW RISK**)<br><br>Veneri et al. (2025): Missing data in this study likely from method-dependent and site-specific sensitivity differences, not selective omission; early samples lacked solid-fraction testing (the only method yielding positives), leading to systematic under-detection rather than directional distortion. **(MODERATE RISK OF BIAS**)

		Ma et al. (2025): No wastewater samples were missing or omitted from analysis, no PCR inhibition occurred (LOD = 10 copies/mL), confirming assay reliability. Missing serum sequences likely reflected low viral titers or RNA degradation, not selective exclusion. Whole-genome sequencing success differed by matrix (serum > urine > wastewater), but all positives were reported, including partial reads. Failures were due to technical degradation, not selective omission. This would reduce precision but not bias the direction of association. → Missing sequencing data: expected random loss due to RNA instability, not bias (**LOW RISK OF BIAS**).
Bias in measurement of outcomes (DOMAIN 6)	Bias introduced by either differential or non-differential errors in measurement of outcome data. Such bias can arise when outcome assessors are aware of intervention status, if different methods are used to assess outcomes in different intervention groups, or if measurement errors are related to intervention status or effects	Refer to results of QA/QC to answer this question for example, replication, internal controls and recovery control.<br><br>Thakali et al. (2022): PMMoV was used to determine the presence of PCR inhibitors and those samples had those were removed from further analysis. PMMoV was used as process control to assess viral recovery were detected in all tested samples. **(LOW RISK)**<br><br>Araujo et al. (2024): No information on measurement of outcome differs by intervention. Whole genome sequencing was performed and the outcome of the detection of DENV is not known prior to intervention.**(MODERATE RISK)**<br><br>Roldan-Hernandez et al. (2024): Measurement of outcome were standardized with accurate results for positive and negative controls. BcoV recoveries were not statistically different in solid and liquid fractions **(LOWRISK)**<br><br>Wolfe et al. (2024): The measurement of outcome were replicated (6 per sample) , with positive and negative controls showed positive and negative result respectively and internal recovery using PMMoV close to 100% . **(LOW RISK)**<br><br>Monteiro et al. (2024): Validation of the results and assays chosen was done in multiple stages. Additionally the methods has been properly assessed through quality control (in detection and quantification). Positive and negative control yielded anticipated outcome **(Low risk)**

		Chandra et al. (2023):The prior knowledge does not influence the measurement **(LOW RISK)**<br><br>Chen at al. (2023): Different method will have different recovery efficiency depending on the measurement of the recovery rate(**MODERATE RISK**)<br><br>Chandra et al. (2021):Measurement of the decay rate is different by the detection method (**MODERATE RISK**)<br><br>Veneri et al. (2025): All outcomes were measured objectively using standardized molecular assays. (**LOW RISK OF BIAS)**<br><br>Ma et al. (2025): Outcomes were measured using validated, standardized RT-qPCR and sequencing methods with appropriate controls (positive, negative, and process spikes). Although laboratory staff knew sampling sites, molecular quantification and sequence confirmation minimize subjective influence. Any measurement error would be random (due to RNA stability), not systematic. (**LOW RISK OF BIAS**)
Bias in selection of the reported result (DOMAIN 7)	Selective reporting of results in a way that depends on the findings and prevents the estimate from being included in a meta-analysis (or other synthesis)	Bias reported results:<br>In general, we expect bias to be introduced in selective reporting of negative results, implying that negative results are more likely to not be published and may skew the outcome of the systematic review in an over-positive direction.<br><br>Thakali et al. (2022):All the tested samples were negative for DENV. Result were reported accoding to analysis plan. **(LOW RISK)**<br><br>Araujo et al. (2024): Total samples were collected 60 samples. However, 56 samples were proceeded for whole genome sequencing (no information for this reduction of sample). This sequencing provided multiple outcome in which DENV one of that. **(SERIOUS RISK)**<br><br>Roldan-Hernandez et al. (2024): All results were reported for both liquid and solid samples **(LOW RISK)**

		Wolfe et al. (2024):The results for all 112 samples were reported including the detection of serotype 3 and non-detection of certain serotypes for all sample. **(LOW RISK).**<br><br>Monteiro et al. (2024): All results was reported based on analysis plan **(LOW RISK)**<br><br>Chandra et al. (2023):Virus analysis was conducted for for different intervention and the result reported according to the analysis plan **(LOW RISK)**<br><br>Chen at al. (2023): There is no deviation in the statistical analysis(**LOW RISK**)<br><br>Chandra et al. (2021): The decay was measured accordingly(**LOW RISK**)<br><br>Veneri et al. (2025): All results were reported according to the analysis plan, with all tested methods and outcomes transparently presented, no selective reporting. (**LOW RISK OF BIAS**)<br><br>Ma et al. (2025):the study adhered to a clear, pre-determined analytical plan comparing concentration methods, validating detection accuracy, and confirming the environmental signal via sequencing and explicitly reported both the overall successful outcomes (early warning, zero false positives) and the technical failures (substantially lower sequencing read counts due to RNA degradation)**(LOW RISK OF BIAS).**

Overall Risk of Bias		Thakali et al. (2022): **SERIOUS RISK**<br>Araujo et al. (2024): **CRITICAL RISK**<br>Roldan-Hernandez et al. (2024): **LOW RISK**<br>Wolfe et al. (2024): **SERIOUS RISK**<br>Monteiro et al. (2024): **LOW RISK**<br>Chandra et al. (2023): **LOW RISK**<br>Chen at al. (2023):**MODERATE RISK**<br>Chandra et al. (2021): **MODERATE RISK**<br>Veneri et al. (2025): **SERIOUS RISK OF BIAS**<br>Ma et al. (2025):**SERIOUS RISK OF BIAS**
