# Supplementary material for: Methodological Approaches to Dengue Virus Detection in Wastewater: A Systematic Review and Meta-Analysis of Positivity Rate
Source: Viruses. 2026 Apr 30;18(5):531. doi: 10.3390/v18050531 (PMC13211638; doi:10.3390/v18050531)
Supplement: Supplementary file 1 [file viruses-18-00531-s001.zip › SUPPLEMENTARY S5_ Assessment of Risk of Bias according to ROBINS-I V2.pdf]

Table S5: Assessment of Risk of Bias according to ROBINS-I V2

Citation	Bias due to confounding (Domain 1)	Bias in classification of intervention (Domain 2)	Bias in selection of participants (Domain 3)	Bias due to deviations from intended interventions (Domain 4)	Bias due to missing outcome data (Domain 5)	Bias in outcomes measurement (Domain 6)	Bias in selection of results reported (Domain 7)	Overall risk of bias
Thakali et al. (2022)	Low risk	N/A	Serious risk	N/A	Low risk	Low risk	Low risk	**Serious Risk**
Araujo et al. (2024)	Critical risk	N/A	Serious risk	N/A	Low risk	Moderate risk	Serious risk	**Critical Risk**
Roldan-Hernandez et al. (2024)	Low risk	Low risk	Low risk	N/A	Low risk	Low risk	Low risk	**Low Risk**
Wolfe et al. (2024)	Lowrisk	N/A	Serious risk	N/A	Low risk	Low risk	Low risk	**Serious Risk**
Monteiro et al. (2024)	Low risk	N/A	Low risk	Low risk	Low risk	Low risk	Low risk	**Low Risk**
Chandra et al. (2023)	Low risk	Low risk	Low risk	Low risk	Low risk	Low risk	Low risk	**Low Risk**
Chen at al. (2023)	Low risk	Low risk	Low risk	Low risk	Low risk	Moderate risk	Low risk	**Moderate Risk**
Chandra et al. (2021)	Low risk	Low risk	Low risk	Low risk	Low risk	Moderate risk	Low risk	**Moderate Risk**
Veneri et al. (2025)	Moderate risk	Low risk	Moderate risk	Low risk	Moderate risk	Low risk	Low risk	**Serious risk**

Ma et al. (2025)	Serious risk	Serious risk	Low risk	Moderate risk	Low risk	Low risk	Low risk	**Serious risk**
