# Supplementary material for: Methodological Approaches to Dengue Virus Detection in Wastewater: A Systematic Review and Meta-Analysis of Positivity Rate
Source: Viruses. 2026 Apr 30;18(5):531. doi: 10.3390/v18050531 (PMC13211638; doi:10.3390/v18050531)
Supplement: Supplementary file 1 [file viruses-18-00531-s001.zip › SUPPLEMENTARY S6_Hand Search - search strategy and process.pdf]

Table S6: Hand Search - search strategy and process

Scheme	Query	PubMed (date)	SCOPUS (date)	Embase (date)	WOS
#1	"Waste Water" OR "Sewage" OR "Wastewater" OR "wastewater treatment plant*" OR "WBE" OR “wastewater-based” OR “sludge” OR “untreated wastewater”	128,957	391,891	168,374	275,011
#2	“surveillance” OR “monitoring” OR “epidemiology” OR “tracking” OR “tracing” OR “wastewater surveillance” OR “wastewater-based surveillance” OR “wastewater-based epidemiology”	1,301,480	2,803,963	1,804,783	1,550,223

#3	"Dengue" OR "Dengue virus RNA" OR "Arthropod-Borne" OR "Arbovirus" OR "Dengue virus*" OR "Human arboviral disease" OR "DENV"	36,236	41,239	46,479	27,142
#4	#1 AND #2 AND #3	22	44	21	19
1st Jan-June 2025	#1 AND #2 AND #3	0	4	8	0

From 1st Jan until october 2025 - no new articles related to dengue WW based on title screening.

Total articles : 118

Duplicates: 32

Articles for title/text screening: 86
